# Berberine Inhibits Proliferation and Down-Regulates Epidermal Growth Factor Receptor through Activation of Cbl in Colon Tumor Cells

**DOI:** 10.1371/journal.pone.0056666

**Published:** 2013-02-14

**Authors:** Lihong Wang, Hailong Cao, Ning Lu, Liping Liu, Bangmao Wang, Tianhui Hu, Dawn A. Israel, Richard M. Peek, D. Brent Polk, Fang Yan

**Affiliations:** 1 Cancer Center, Xiamen University Medical College, Xiamen, People's Republic of China; 2 Department of Pediatrics, Division of Gastroenterology, Hepatology and Nutrition, Vanderbilt University Medical Center, Nashville, Tennessee, United States of America; 3 Department of Medicine, Tianjin Medical University General Hospital, Tianjin, People's Republic of China; 4 Department of Breast Cancer Medical Oncology, Key Laboratory of Breast Cancer Prevention and Therapy, Tianjin Medical University Cancer Institute and Hospital, Tianjin, People's Republic of China; 5 Department of Medicine, Vanderbilt University Medical Center, Nashville, Tennessee, United States of America; 6 Department of Cancer Biology, Vanderbilt University Medical Center, Nashville, Tennessee, United States of America; 7 Departments of Pediatrics and Biochemistry and Molecular Biology, University of Southern California and Saban Research Institute of Children's Hospital Los Angeles, Los Angeles, California, United States of America; University of Central Florida, United States of America

## Abstract

Berberine, an isoquinoline alkaloid, is an active component of Ranunculaceae and Papaveraceae plant families. Berberine has been found to suppress growth of several tumor cell lines in vitro through the cell-type-dependent mechanism. Expression and activation of epidermal growth factor receptor (EGFR) is increased in colonic precancerous lesions and tumours, thus EGFR is considered a tumour promoter. The aim of this study was to investigate the effects and mechanisms of berberine on regulation of EGFR activity and proliferation in colonic tumor cell lines and in vivo. We reported that berberine significantly inhibited basal level and EGF-stimulated EGFR activation and proliferation in the immorto Min mouse colonic epithelial (IMCE) cells carrying the *APC* ^min^ mutation and human colonic carcinoma cell line, HT-29 cells. Berberine acted to inhibit proliferation through inducing G1/S and G2/M cell cycle arrest, which correlated with regulation of the checkpoint protein expression. In this study, we also showed that berberine stimulated ubiquitin ligase Cbl activation and Cbl's interaction with EGFR, and EGFR ubiquitinylation and down-regulation in these two cell lines in the presence or absence of EGF treatment. Knock-down Cbl expression blocked the effects of berberine on down-regulation of EGFR and inhibition of proliferation. Furthermore, berberine suppressed tumor growth in the HT-29 cell xenograft model. Cell proliferation and EGFR expression level was decreased by berberine treatment in this xenograft model and in colon epithelial cells of *APC* ^min/+^ mice. Taken together, these data indicate that berberine enhances Cbl activity, resulting in down-regulation of EGFR expression and inhibition of proliferation in colon tumor cells.

## Introduction

Berberine is an active component of Ranunculaceae and Papaveraceae families of plant. It represents one of well-studied naturally occurring isoquinoline alkaloids. Studies have revealed berberine's pharmacodynamic effects on clinical uses, such as treating gastrointestinal diseases, including bacterium and virus-associated diarrhea and acute gastroenteritis, and parasitic infections, type II diabetes, hypertension, and arrhythmia [1]. Increasing evidence has demonstrated berberine's immunoregulatory effects on inflammation. For example, berberine promotes recovery of dextran sulfate sodium-induced intestinal injury and inflammation in mice, which correlates with its inhibitory effects on inflammatory cytokine production by colonic macrophages and epithelial cells through suppressing signaling pathways [2].

Recent research focused on berberine's anti-tumor effect has shown that berberine inhibits growth of broad tumor cell types derived from leukocytes, liver, lung, gastrointestinal tract, oral, skin, brain, bone, bladder, breast, cervix, and prostate [3]. Several mechanisms involved in berberine's antitumor activity have been identified, which include stimulating caspase-dependent apoptosis and caspase-independent cell death by activation of apoptosis-inducing factor, suppressing cancer cell growth and proliferation by induction of cell cycle arrest, and inhibiting metastasis by down-regulating matrix metalloproteinases [1]. Berberine-regulated signaling pathways involved in its anti-cancer effects have been found, which include p53, MAPK, and NF-κB [3], [4]. The findings that mechanisms underlying berberine's anti-cancer effects are different among tumor cell types indicate the cell type specific effect of berberine on inhibition of tumor development.

Epidermal growth factor receptor (EGFR) belongs to the ErbB family of type 1 transmembrane receptor tyrosine kinases. The cytoplasmic domain of EGFR contains the kinase domain as well as tyrosine residues which are themselves targets of EGFR- and other kinase-mediated phosphorylation. Although EGFR is activated directly by various ligands, including EGF, transforming growth factor-α, amphiregulin, EGFR can also be indirectly transactivated in response to stimuli such as tumour necrosis factor [5]. Ligation of EGFR leads to receptor dimerization, phosphorylation and increased tyrosine kinase activity [6].

Following ligand binding to EGFR, the Cbl ubiquitin ligase, a family of E3 ubiquitin ligases, is tyrosine phosphorylated by the receptor, which requires prior EGFR phosphorylation on tyrosine 1045 and/or serines 1046/1047 [7], or indirectly through interaction with the adaptor protein growth factor receptor-bound protein 2 (GRB2) to promote EGFR ubiquitinylation at the plasma membrane [8]. Tyrosine phosphorylation of Cbl increases its ubiquitin ligase activity for ubiquitinylation of EGFR on multiple sites [9], resulting in translocation of EGFR to the endosomal compartment [10]. EGFR is subsequently sorted by incompletely understood mechanisms through distinct vesicles for either recycling to the plasma membrane or destruction [11], [12].

EGFR activity is essential for development and insufficient EGFR signaling causes death in the perinatal period. EGFR regulates multiple aspects of colon epithelial homeostasis including proliferation, cell survival, wound closure, and barrier function in order to optimize epithelial responses to injury. However, excessive EGFR signaling is observed in many human cancers of epithelial origin, which is involved in neoplastic transformation, including initiation and progression of cancers [13], [14]. EGFR expression or activation is increased in many colonic precancerous lesions and tumours [15], [16] and is implicated in numerous animal models of gastrointestinal tumourigenesis [16], [17], [18]. Therefore, EGFR is one of key targets of the therapeutic strategy designed to treat cancers. Currently, two EGFR monoclonal antibodies have been approved for the treatment of metastatic colorectal cancer (cetuximab and panitumumab) [19]. But the currently used therapeutic agents are not effective against all receptor-positive tumors and new therapies with maximal disruption of EGFR oncogenicity are needed.

The aim of this study was to investigate the effects and mechanisms of berberine on regulation of EGFR and proliferation in colon tumor cells. We report that tumor growth in the HT-29 cell xenograft model was suppressed by berberine. Cell proliferation and EGFR expression level was decreased by berberine treatment in this xenograft model and in colon epithelial cells of *APC* ^min/+^ mice. Furthermore, berberine stimulated Cbl activation, which mediated down-regulation of EGFR and inhibition of cell proliferation in mouse and human colon tumor cells. These results reveal a novel mechanism of berberine's function and provide insight into application of berberine for colon cancer therapy.

## Results

### Berberine downregulates EGFR expression and activates Cbl in colon tumor cells

**Figure 1:**
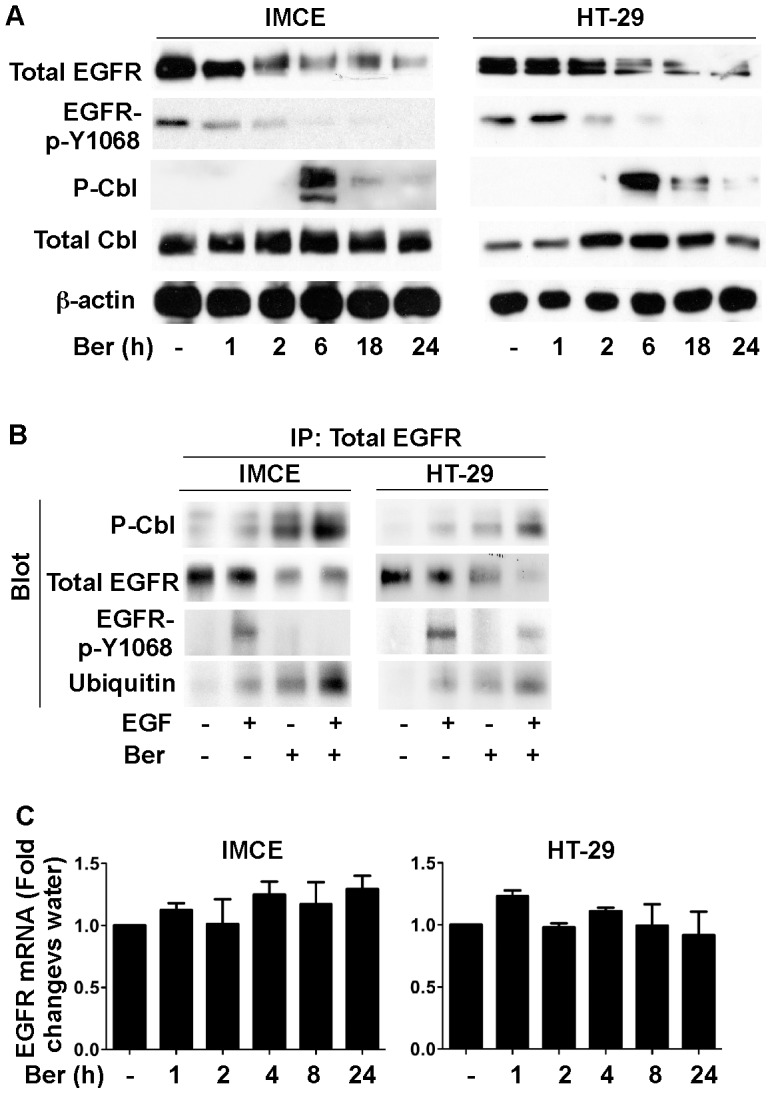
Berberine down-regulates EGFR level and activates Cbl in colon tumor cells. (A) cells were cultured in serum-starved RPMI 1640 medium at 37°C (non-permissive condition) for 24 hours with or without berberine at 50 µM treatment for indicated times from the end of the experiment. Cellular lysates were collected for Western blot analysis to detect indicated signaling pathways. (B) cells were treated with berberine at 50 µM under non-permissive condition for 18 hours in the presence or absence of EGF (30 ng/ml) treatment for 5 minutes. 1 mg of cellular lysates were prepared for immunoprecipitation using an anti-EGFR antibody. EGFR and co-immunoprecipated proteins were analyzed using Western blot analysis. The β-actin blot was used as a protein loading control. (C) mRNA was isolated from cells cultured under non-permissive condition for 24 hours with or without berberine at 50 µM treatment for indicated times from the end of the experiment. Real-time PCR analysis was performed to detect the EGFR mRNA level. The EGFR mRNA expression level in the control group was set as 100%, and mRNA expression levels in treated groups were compared to the control group. Data in this Figure are representative of three separate experiments.

To determine the effects of berberine on regulation of EGFR activity in colon cancer cells, we chose two colonic tumor cell lines, the immorto Min mouse colonic epithelial (IMCE) cells carrying the *APC* ^min^ mutation [20], and HT-29 cells which were isolated from human colonic carcinoma. In these two cell lines, we detected EGFR phosphorylation on the tyrosine autophosphorylation site 1068 under the condition without any treatment (Figure 1A), which suggests that EGFR is activated in these two cell lines. Addition of berberine resulted in decreasing the total EGFR and phosphorylated EGFR levels in both IMCE and HT-29 cells (Figure 1A). In addition to regulation of basal levels of EGFR expression and activation, berberine was able to down-regulate EGFR expression in IMCE and HT-29 cells treated with EGF (Figure 1B). Furthermore, EGF-stimulated EGFR activation was also inhibited by berberine in these two cell lines (Figure 1B).

Since tyrosine phosphorylation of Cbl increases its ubiquitin ligase activity for ubiquitinylation of EGFR [9], resulting in translocation of EGFR to the endosomal compartment and degradation [10], we determined the effect of berberine on Cbl activation. Berberine increased Cbl phosphorylation in both IMCE and HT-29 cells (Figure 1A). Furthermore, immunoprecipation using anti-EGFR antibody showed that berberine increased phosphoryleted Cbl interaction with EGFR and EGFR ubiquitinylation in both of the presence and absence of EGF exposure (Figure 1B). These data suggest that berberine up-regulation of Cbl activity may mediate EGFR down-regulation in colon tumor cells.

To exam whether berberine has any effects on transcriptional regulation of EGFR, we employed real-time PCR analysis to detect the EGFR mRNA level in IMEC and HT-29 cells treated with berberine. We found that berberine did not affect the EGFR messenger level in these two cell lines (p>0.05, Figure 1C). In consistent to this finding, data from our Affymetrix microarray showed that the EGFR gene expression level was not changed in IMEC cells treated with berberine (Figure S1). Therefore, enhancing EGFR degradation is a potential mechanism by which berberine inhibits EGFR's function in colon tumor cells.

### Berberine inhibits proliferation in colon tumor cells through inducing cell cycle arrest G1/S and G2M phase

**Figure 2:**
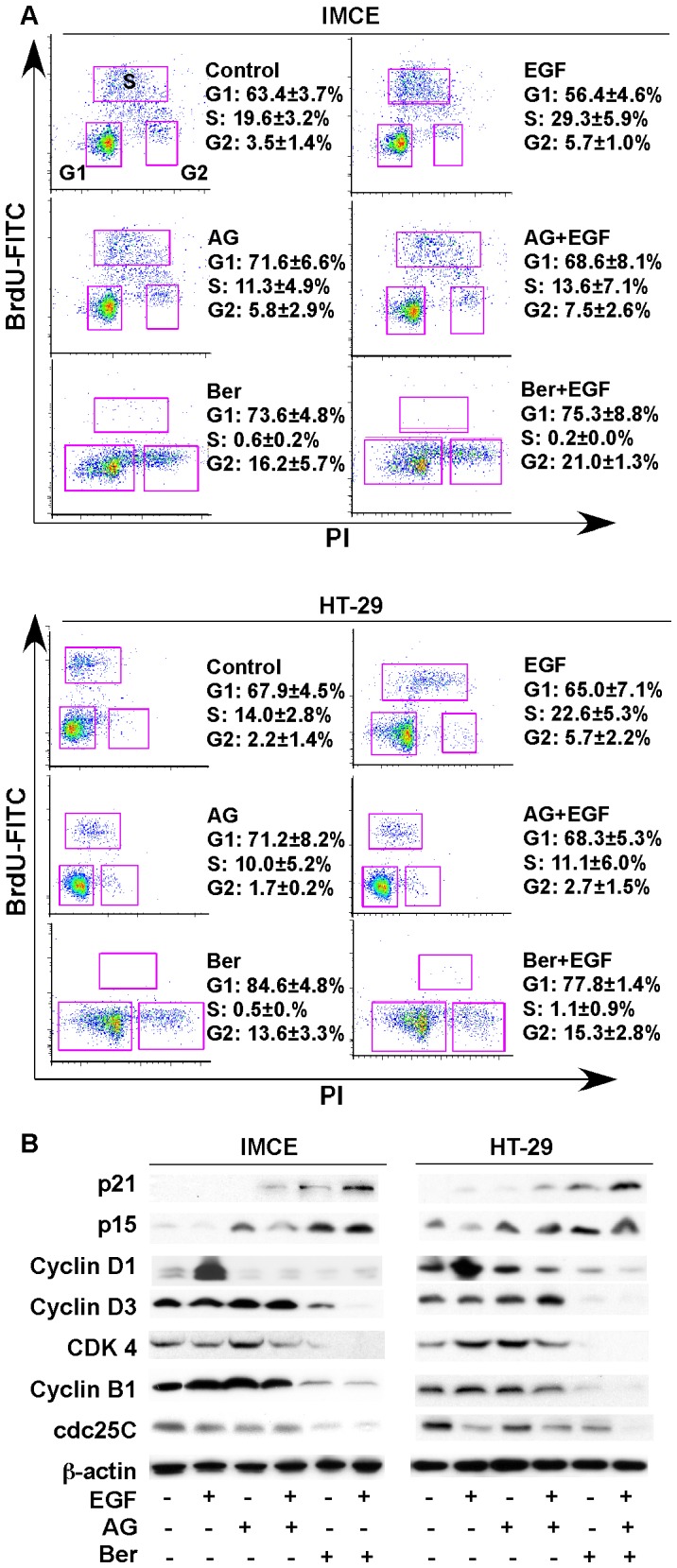
Berberine inhibits basal and EGF-stimulated proliferation in colon tumor cells. (A) cells were treated with berberine at 50 µM under non-permissive condition for 24 hours in the presence or absence of EGF (30 ng/ml) treatment for 24 hours. Cell proliferation was detected by BrdU labeling and staining with FITC-conjugated anti-BrdU antibody and propidium iodide (PI) staining for flow cytometry. Density plots with BrdU-FITC vs PI and percentages of cells in G1, S, and G2 phase are shown. (B) cells were treated as described in (A). Cellular lysates was prepared for Western blot analysis to detect expression of the cell cycle checkpoint proteins. Data in this Figure are representative of three separate experiments.

We have reported that berberine inhibited cell growth in a concentration and time-dependent manner, and that berberine induced caspase-independent colon tumor cell death [21]. In addition to cell death, decreased proliferation is another factor contributing to cell growth inhibition. EGFR, which has been identifies as one of factors to promote tumor cell proliferation was inhibited by berberine (Figure 1). Thus, we next investigated berberine's effect on cell proliferation. Cell proliferation is generally controlled by the progression of three distinctive phases (G0/G1, s and G2/M) of the cell cycle, and cell cycle arrest is considered one of the most common causes of the inhibition of cell proliferation. Cells were treated with EGF in the presence or absence of berberine or an EGFR kinase inhibitor, AG1478, and labeled with BrdU and PI to detect cell cycle progression by flow cytometry. EGF-stimulated cells were found at higher numbers in S phase, compared with controls. Berberine significantly reduced cells in S phase and increased cells in G2 phase in the absence or presence of EGF exposure in these two cell lines (Figure 2A). AG1478 treatment decreased cells in S phase in both IMCE and HT-29 cells with or without EGF stimulation (Figure 2A). These data indicate that berberine induced cell cycle arrest in G1/S and G2/M phase. Since inhibition of EGFR by AG1478 only induced cell cycle arrest in G1/S phase, berberine-induced cell cycle arrest in G2/M phase may be through other signaling pathways. Thus, berberine inhibits proliferation in colon tumor cells through multiple mechanisms.

As we previously reported that berberine induces apoptosis-inducing factor (AIF)-mediated cell death, but not caspase-dependent apoptosis [21], we did not find activation of PARP and caspase-3 by berberine in IMEC and HT-29 cells in this study (Figure S2). Therefore, berberine regulated caspase-independent cell death and inhibition of proliferation may serve as two mechanisms for inhibition of colon tumor growth.

Cyclins and cyclin-dependent kinase (CDKs) are cell cycle regulators and the formation of cyclin-CDK complexes trigger their functions. The function of cyclin-CDK complexes is inhibited by CDK inhibitors. To further examine whether berberine-induced cell cycle arrest was associated with the expression of cell cycle regulators, Western blot analysis of cellular lysates was performed. The data showed that berberine up-regulated CDK inhibitors, p21 and p15 in both IMCE and HT-29 cells (Figure 2B). In addition, berberine decreased cell cycle regulators which mediated the transition from G1 to S phase, cyclin D1, D3, and CDK4, and G2 to M phase, cyclin B1 and cell division cycle (cdc) 25C (Figure 2B). Consistent to the data that AG1478 induced G1/S phase cell cycle arrest (Figure 2A), inhibition of EGFR by AG1478 up-regulated CDK inhibitors, p21 and p15, and down-regulated cyclin D1 and CDK4, with no effects on cyclin B1 and cdc25C (Figure 2B). These findings indicate that berberine induces G1/S and G2/M phase cell cycle arrest by modulating the cell cycle regulators in colon tumor cells.

Cbl is required for berbrine down-regulation of EGFR and inhibition of proliferation in colon tumor cells.

**Figure 3:**
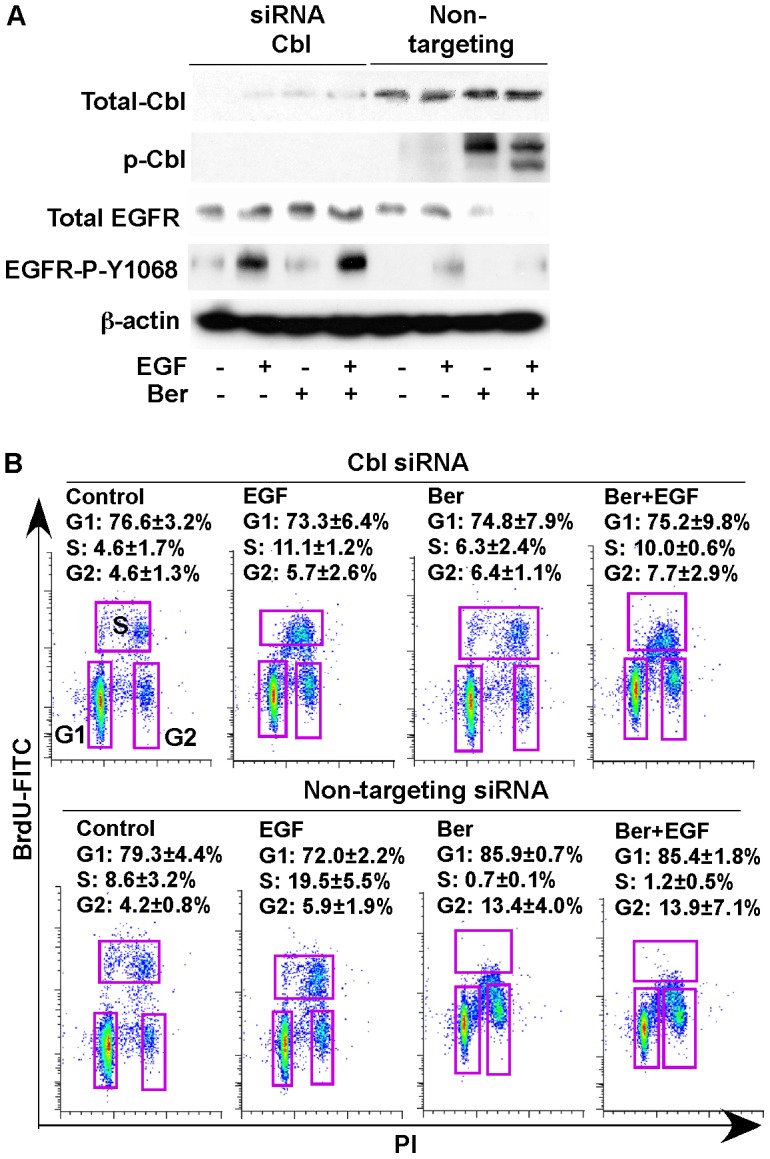
Cbl is required for berberine inhibition of proliferation by berberine in IMCE cells. Cells were transfected with Cbl siRNA or non-targeting siRNA. (A) at the 30-hour of posttransfection, cells were treated with berberine at 50 µM for 18 hours under non-permissive condition in the presence or absence of EGF (30 ng/ml) treatment for 5 minutes. Cellular lysates were collected for Western blot analysis to detect Cbl expression and indicated signaling pathways. (B) at the 24-hour of posttransfection, cells were treated with berberine at 50 µM for 24 hours under non-permissive condition in the presence or absence of EGF (30 ng/ml) treatment for 24 hours to detect cell proliferation, as described in Figure 2A. Density plots with BrdU-FITC vs PI and percentages of cell s in G1, S, and G2 phase are shown. Data in this Figure are representative of three separate experiments.

To further study the role of Cbl activation in berberine-induced down-regulation of EGFR and inhibition of proliferation in colon tumor cells, we knocked down Cbl expression in IMCE cells by transfecting Cbl targeted siRNA (Figure 3A). We found that suppression of Cbl expression blocked berberine's effects on inducing EGFR down-regulation and inhibition of EGFR activation (Figure 3A). Furthermore, berberine induced cell cycle arrest in G1/S and G2/M phase in non-targeting siRNA, but not in Cbl siRNA transfected IMCE cells (Figure 3B). These results indicate the requirement of Cbl for berberine-induced inhibitory effects on EGFR and proliferation in tumor cell lines.

The known function of EGFR ubiquitylation is for endosomal receptor sorting and lysosomal degradation [22]. Thus, to further assess the role of berberine-stimulated EGFR ubiquitination leading to EGFR degradation on inhibition of proliferation, chloroquine, a lysosomal protease inhibitor, was used to inhibit ubiquitinated EGFR degradation in lysosome. Chloroquine blocked berberine induced EGFR down-regulation in IMEC and HT-29 cells (Figure S3). Furthermore, the inhibitory effect of berberine on cell proliferation, detected by increased p21 expression in these two cell lines was abolished by co-treatment with chloroquine (Figure S3). These results suggest that ubiquitination of EGFR leading to the receptor down-regulation by berberine plays a role in inhibition of colon tumor cell proliferation.

### Berberine enhances EGFR down-regulation and inhibits proliferation in colonic epithelial cells in *APC* ^min/+^ mice

**Figure 4:**
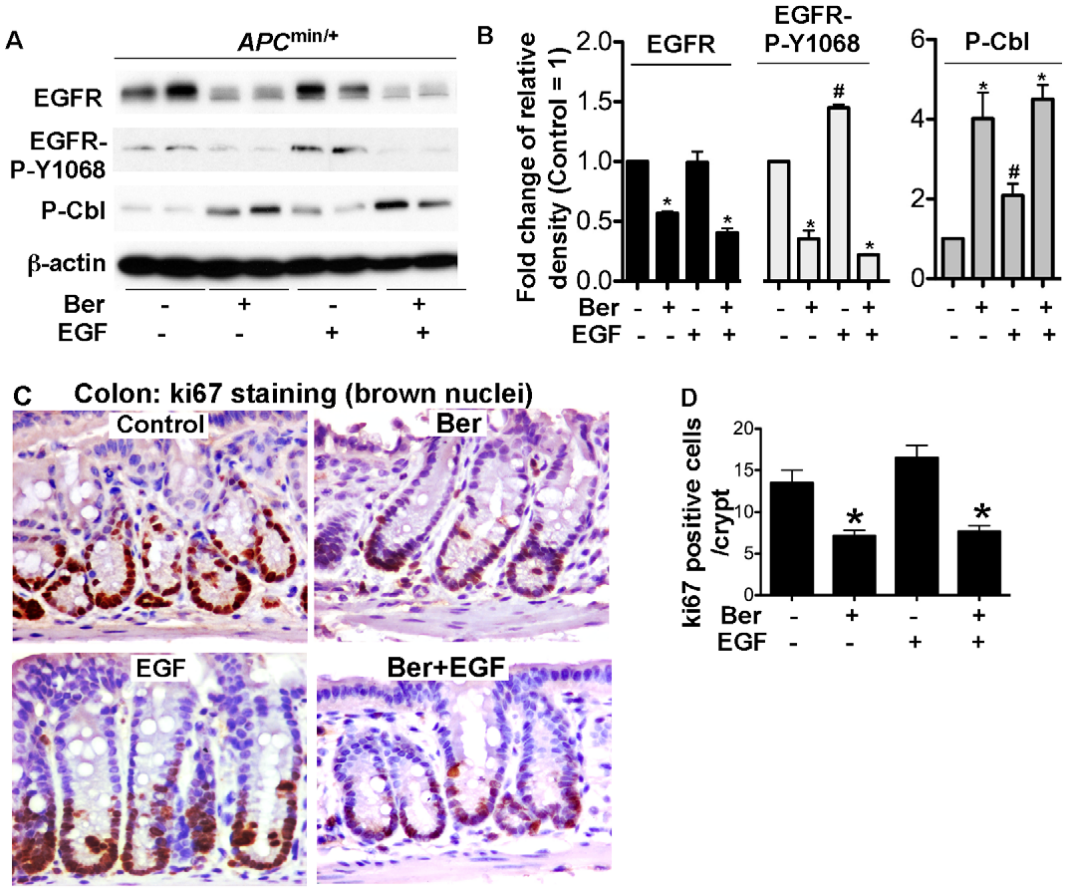
Berberine induces EGFR down-regulation and inhibits proliferation in colonic epithelial cells in *APC* ^min/+^ mice. 5–6 week old mice were gavaged with berberine at 100 mg/kg bodyweight for 3 days with or without EGF injection for 2 hours before sacrificed. (A–B) Colonic epithelial cells were collected for Western blot analysis to detect the indicated signaling pathways. The protein band ratio is calculated by comparing the relative density of the protein band on Western blots to that of β-actin band from the same mouse. The average ratio in control was set as 100%, the fold change of the ratio in treated mice is shown. (C–D) colon tissue was fixed and prepared for immunohistochemistry of ki67. Brown nuclei represent ki67 positive staining. Ki67 positive staining cells were counted in at least 100 crypts. The number of ki67 positive staining cells per crypt is shown. N = 5 mice in each treatment group.

**Figure 5:**
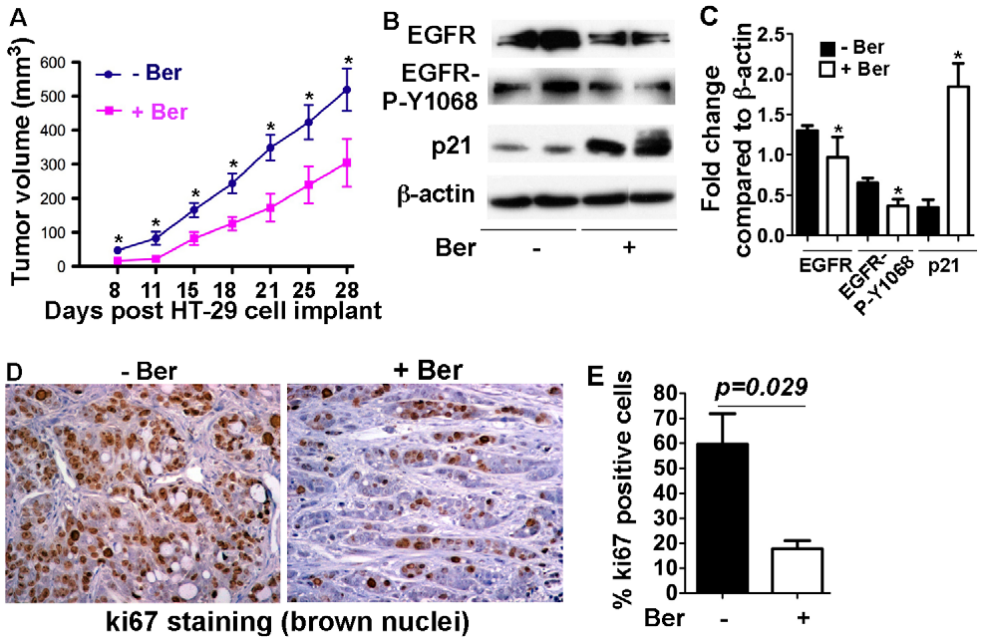
Berberine exhibits an anti-tumor effect on the xenograft model. Nude mice bearing HT-29 xenografts were treated with berberine (0.1% in drinking water) starting at the day of inoculation until the end of the experiment. Tumor volumes were measured at the indicated days post HT-29 cell implant (A). Tumor tissues were prepared for Western blot analysis of indicated signaling pathways. Anti-β-actin antibody was used as a protein loading control (B). The fold change of the band density was determined by comparing the density of indicated band to the β-actin band of the same mouse (C). Tumor tissues were fixed and prepared for immunohistochemistry of ki67. Brown nuclei represent ki67 positive staining (D). The percentage of ki67 positive staining cells was quantitatively analyzed using Ariol SL-50 automated slide scanner (E). In C, * p<0.05 compared the control mice. In D, * p<0.01 compared the corresponding group in control mice. N = 7 mice in each group.

Our *in vitro* data indicate that berberine plays an anti-proliferative role and down-regulates EGFR in colon tumor cells. To determine whether these effects could be extended *in vivo*, *APC* ^min/+^ mice at 4–5 week old were gavaged with berberine for 3 days with or without EGF injection for 2 hours before sacrificed. Colonic epithelial cells were isolated for Western blot analysis and tissue was fixed and prepared for immunohistochemistry. We found EGFR Y1068 phosphorylation in colonic epithelial cells from *APC* ^min/+^ mice without EGF treatment (Figure 5A). Berberine decreased both EGFR expression and phosphorylation in colonic epithelial cells from untreated mice. Berberine also inhibited EGF-stimulated EGFR activation (Figure 4A–B). Importantly, berberine stimulated Cbl phosphorylation under the condition in the presence or absence of EGF treatment (Figure 4A–B).

To determine if berberine exerts an anti-proliferative effect *in vivo*, cell proliferation was detected by ki67 immunohistochemistry. Berberine significantly reduced colonic epithelial cell proliferation in untreated *APC* ^min/+^ mice (Figure 4C–D). EGF-stimulated cell proliferation was also inhibited by berberine (Figure 4C–D). Thus, these data indicate that berberine suppression of EGFR function may mediate its anti-proliferative effects on colonic epithelial cells in *APC* ^min/+^ mice.

### Berberine inhibits tumor growth in colon cancer xenograft model

We next examined the in vivo effects of berberine on colon tumor growth using the HT-29 colon cancer xenograft model. Immuno-deficient Nude mice bearing HT-29 xenografts were treated with berberine in drinking water beginning at the day of inoculation until the end of the experiment. Compared to the control mice, berberine treatment significantly decreased the tumor growth (Figure 5A). EGFR expression and activation levels were decreased in tumor cells of mice treated with berberine (Figure 5B and C). ki67 immunohistochemistry revealed a significant decrease in cell proliferation in tumors from berberine treated mice (Figure 5D and E). Furthermore, berberine up-regulated p21, suggesting cell cycle arrest induced by berberine in the tumor cells. These results further suggest the inhibitory effects of berberine on colon tumor cell proliferation, which is associated with inhibition of EGFR function.

## Discussion

Considerable attention has been given to the identification of naturally occurring chemopreventive or chemotherapeutic reagents capable of inhibiting or reversing the tumor development process, including colorectal cancer. Berberine has shown its anti-tumor effects on broad types of tumor cells through different mechanisms [1]. The present study demonstrated the potent inhibitory activity of berberine for proliferation through inducing G1/S and G2/M cell cycle arrest in colon tumor cells. Although berberne has been reported to induce cell cycle arrest before, such as G1/S phase arrest in human epidermoid carcinoma cells [23] and G2/M phase arrest in human carcinoma cells [24], no mechanisms have been provided in the previous studies. We provided a plausible mechanism of berberine's action, which involves stimulation of Cbl activation, thus enhancing EGFR down-regulation, leading to inhibition of proliferation in colon tumor cells. However, berberine does not affect the EGFR gene expression level.

One of the significant roles of EGFR signaling is to promote cell proliferation. We showed that an EGFR kinase inhibitor, AG1478, induced cell cycle arrest in G1/S phase in both IMCE and HT-29 cells. However, berberine induced cell cycle arrest in G1/S and G2/M phase. These data suggest that, in addition to inhibition of EGFR, berberine should exert other mechanisms to induce cell cycle arrest. Therefore, we performed gene microarray analysis to find more targets of berberine for regulation of cell proliferation. We found a gene, Gadd45α, which was up-regulated by berberine in the colon tumor cell line. Gadd45α belongs to Gadd45 family of genes, which are induced in response to multiple environmental and physiological stresses [25]. Gadd45α has been shown to interact with the cdc2/cyclin B1 complex to inhibit the kinase activity of the complex, thereby resulting in G2/M cell cycle arrest [26], [27]. In addition, berberine regulated expression of other two genes involved in inducing G1/S phase arrest. Berberine up-regulated DDIT3, a transcription factor promoting G1/S cell cycle arrest. Expression of DDIT3 is enhanced in response to a variety of cellular stress, notably ER stress [28]. Another gene, EGLN3 (also called prolylhydroxyltion domain containing enzyme 3, PHD3) was down-regulated by berberine. Depletion of PHD3 under hypoxia has been shown to lead to cell cycle arrest at the G1/S phase and reduce the amount of hyperphosphorylated retinoblastoma protein and the amount of cyclin D1. Moreover, PHD3 inhibition increases the expression of cyclin-dependent kinase inhibitor p27 [29].

A significant finding from the present study is that berberine down-regulates EGFR through activation of Cbl and increasing Cbl interaction with EGFR. Signaling pathways that are activated by ligand binding to cell surface receptors are responsible for determining many aspects of cellular function and fate. Although this outcome is primarily determined by the nature of the ligand and its receptor, it is essential that intracellular enzymes, adaptor proteins and transcription factors are correctly assembled to convey the intended response. It has been well studied that EGFR phosphorylation on tyrosine 1045 is thought to be involved in EGF-mediated Cbl tyrosine phosphorylation [7] to increase its ubiquitin ligase activity for ubiquitinylation of EGFR [9], leading to either recycling to the plasma membrane or destruction by incompletely understood mechanisms [11], [12]. However, in our studies, EGFR tyrosine 1045 phosphorylation was decreased at the same level as EGFR tyrosine 1068 in IMCE and HT-29 cell lines treated with berberine (data not shown), which indicates that berberine regulation of Cbl is independent of EGFR 1045 phosphorylation. Since we have reported that berberine stimulates ROS production in colon tumor cells to stimulate caspase-independent cell death [21], we have tested the role of ROS scavenger on berberine regulation of EGFR and proliferation. We did not find any effects of blocking ROS on EGFR down-regulation, inhibition of proliferation, and Cbl phosphorylation by berberine (data not shown). Thus, although our results reveal a new mechanism for berberine's action on EGFR that may be important for colon tumor prevention and treatment, further studies are needed to elucidate the mechanisms underlying berberine up-regulation of Cbl activation.

Advances in cancer biology have identified multiple signaling pathways involved in the formation and progression of colorectal carcinomas, thus, inhibition of one signaling pathway alone is not sufficient to block cancer cell growth [30]. Berberine has demonstrated multiple actions for inhibition of cancer cell growth. It has been reported that berberine stimulates caspase-dependent apoptosis, through regulating signaling pathways, such as p53, MAPK, and NF-κB [3], [4]. Our previous studies have shown that activation of AIF by berberine leading to caspase-independent cell death and the link between berberine-induced ROS generation and AIF activation in colon tumor cells [21]. Berberine has also been found to induce autophagic cell death and mitochondrial apoptosis in liver cancer cells [31].

Results from the present study provided new findings regarding berberine's anti-tumor effects. Increased EGFR expression and/or activity is related to many human malignancies. Extensive research has provided considerable evidence concerning the molecular mechanisms which provide EGFR degradation. Cbl mediated ubiquitination, endosomal sorting and lysosomal degradation have become well-investigated cornerstones. Thus, the ability of berberine stimulated Cbl-dependent EGFR down-regulation may underscore its importance to suppressing diseases such as cancer. In addition to down-regulation of EGFR, berberine inhibits the expression of the oncogene, Myc. Myc is well known to enhance cellular proliferation, differentiation, and apoptosis. N-myc can support most c-myc functions necessary in mice [32]. Another oncogene, MDM2 has also been reported by other group to be inhibited by berberine [33]. It should be noted that we found berberine up-regulates Sesn2, which mediates inhibition of PI3K-mTOR complex (mTORC)1-axis [34]. Therefore, these results suggest that berberine has the potential to simultaneous target multiple pathways to treat colorectal tumors.

In summary, the present study provides insight into the mechanism of berberine inhibition of cell growth in both human and mouse colon tumor cells. These results will serve as the basis for *in vivo* studies to investigate the effects of berberine on colon tumor cancer treatment.

## Materials and Methods

### Cell culture and treatment

The IMCE cell line was provided by Dr. Robert Whitehead at Vanderbilt University, Nashville, TN (generation and characterization of this cell line was published in [20]). IMCE cell line was generated from the colonic epithelium of F1 Immorto-*APC* ^min/+^ mouse hybrid. The immotomouse is an H-2Kb–tsA58 mouse expressing a heat-labile simian virus 40 large T antigen with an IFN-γ-inducible promoter. Thus, IMCE cells carry both the mutant *APC* ^min^ gene and a temperature-sensitive mutant of the SV40 large T gene. IMCE cells were maintained in RPMI 1640 medium supplemented with 5% heat-inactivated fetal bovine serum (FBS), 5 U/ml of murine IFN-γ, 100 U/ml penicillin and streptomycin, 5 µg/ml insulin, 5 µg/ml transferrin, 5 ng/ml selenous acid at 33°C (permissive condition) with 5% CO2.

HT-29 cells isolated from human colorectal adenocarcinoma (ATCC, HTB-38™) were grown in DMEM medium supplemented with 10% heat-inactivated FBS and 100 U/ml penicillin and streptomycin at 37°C with 5% CO_2_.

IMCE cells were maintained in serum-starved RPMI 1640 medium containing 0.5% FBS and 100 U/ml penicillin and streptomycin (no IFN-γ) at 37°C (non-permissive condition), and HT 29 cells were cultured in serum-starved DMEM medium containing 0.5% FBS and 100 U/ml penicillin and streptomycin at 37°C for 18 hours before treatment. Cells were treated with berberine chloride (Sigma-Aldrich) in the presence or absence of murine EGF (for IMEC), human EGF (for HT-29 cells) (Pepro Tech, Inc.) or chloroquine diphosphate salt (Sigma-Aldrich).

### Transient transfection of siRNA Cbl

Cells were transiently transfected with either 30 nM non-targeting siRNA or 30 nM mouse Cbl siRNA (Santa Cruz Biotechnology, INC) at 80% confluence using Lipofectamine RNAiMAX Reagent (Invitrogen Corporation), according to the manufacturer's instructions. After 36-hour transfection, cells were treated with berberine and EGF for detecting Cbl expression level and signaling by Western blot analysis and cell proliferation by BrdU-labeling.

### Proliferation assay

At the end of treatment, IMCE and HT-29 cells were incubated with BrdU (GE Healthcare UK Limited) at 10 µM in cell culture medium for 1 hour at 37°C. The cells were harvested, washed with phosphate buffered saline (PBS), and fixed in 70% ethanol (vol/vol) in PBS at 2×10^6^ cells/ml solution at 4°C overnight to process for cell cycle analysis. Briefly, cells were incubated in 2N HCl containing 0.5% BSA for 30 min at room temperature and washed with 0.1 M Borax and PBS containing 0.5% BSA. Then cells were labeled with anti-BrdU-FITC antibody (Invitrogen Molecular Probes) for 30 minutes at room temperature in the dark, followed by PI staining (100 µg/ml in PBS containing 20 µg/ml RNase A) for 15 minutes at room temperature in the dark. The cell cycle distribution of cells was analyzed using multi-color flow cytometry equipped with BD LSRII system (BD Biosciences).

### Immunoprecipitation

Cells were lysed in 50 mM Tris (pH 7.4) containing 150 mM NaCl, 0.1% NP40, and protease inhibitor and phosphatase inhibitors (Sigma-Aldrich Corporation) and protein concentrations were determined by the BCA assay (Pierce, Rockford). 1 mg of cellular proteins were incubated with 2 µg of anti-EGFR antibody (Cell Signaling Technology) for 4 hours at 4°C, then were incubated with 30 µl of protein A/G-Agarose beads (Santa Cruz Biotechnology, INC) over night at 4°C. Beads were collected by centrifugation at 1,000 g for 2 min and washed 2 times with lysis buffer containing 1 M NaCl. Proteins were eluted from the beads by boiling in Laemmli sample buffer.

### Real-Time PCR Analysis

Total RNA was isolated from cells using an RNA isolation kit (Qiagen, Valencia, CA) and was treated with RNase-free DNase. Reverse transcription was performed using the High Capacity cDNA Reverse Transcription kit and the 7300 Real Time PCR System (Applied Biosystems, Foster City, CA). The data were analyzed using Sequence Detection System V1.4.0 software. All primers were purchased from Applied Biosystems, human EGFR (Hs01076078) and mouse EGFR (Mm00433023). The relative abundance of glyceraldehyde-3-phosphate dehydrogenase (GAPDH) mRNA was used to normalize levels of the mRNAs of interest. All cDNA samples were analyzed in triplicate.

### Mice, treatment, and colonic epithelial cell isolation

All animal experiments were performed according to a protocol approved by the Institutional Animal Care and Use Committee at Vanderbilt University, Nashville, TN, USA. *APC* ^min/+^ mice on the C57BL/6J background (The Jackson Laboratory) were housed on a 12-h light and 12-h dark cycle. 4–5 week old mice were gavaged with berberine at 100 mg/kg bodyweight/day for 3 days. Mice were then injected with EGF (1 µg/g bodyweight in 200 µl PBS containing 2% BSA) and euthanized 1 hour after injection. Colon tissue was used for isolation of colonic epithelial cells or fixed in 4% paraformaldehyde at 4°C overnight before preparing paraffin-embedded tissue sections.

Colonic epithelial cells were isolated as described before [35]. The colon tissues were incubated with 0.5 mM dithiothreitol and 3 mM EDTA at room temperature for 1.5 hours without shaking. Crypts were released from the colon by vigorous shaking and the suspension was filtered through 70 µm cell filter strainer. Epithelial cells were sorted using a biotin-labeled E-cadherin antibody and streptavidin magnetic beds and washed with PBS.

### Tumor xenograft

2×10^6^ HT-29 cells in 200 µl PBS were subcutaneously inoculated into the flank of 6- to 8-week old Nude mice (Charles River Laboratories). Mice were euthanized after 28 days after inoculation. Berberine in drinking water (0.1%) was administered to mice starting at the day of inoculation until the end of the experiment. Tumor volumes were calculated by measuring two perpendicular diameters and by using the formula of V = 0.5×a×b^2^ where a and b are the larger and smaller diameters, respectively. Tumor tissues were lysed or fixed in 4% paraformaldehyde at 4°C overnight before preparing paraffin-embedded tissue sections.

### Preparation of cellular and tissue lysates for Western blot analysis

HT29 and IMCE cells and colonic epithelial cells were solublized using cell lysis buffer containing 10 mM Tris (pH 7.4), 1% Triton X-100, 1 mM EDTA, 1 mM EGTA, 150 mM NaCl, and protein inhibitor cocktail (1∶100) and incubated for 1 hour on ice. Xenograft tissues were solubilized in CellLytic™ MT mammalian tissue lysis/extraction reagent (Sigma-Aldrich Corporation) and homogenized using TissueLyser (Qiagen). The protein concentration was determined using a BCA protein assay kit (Thermo Fisher Scientific). The cellular lysates were mixed with Laemmli sample buffer and boiled for 10 minutes.

Proteins were separated by SDS-PAGE for Western blot analysis using antibodies against cell cycle regulation proteins, p21, p18, p15, p27, cyclins B, D1, D3, CDK2, and cdc25c (Cell Signaling Technology), and anti-EGFR, anti-phospho-EGFR Y1068, anti-phospho-Cbl, (Cell Signaling Technology), anti-Cbl (Santa Cruz Biotechnology), anti-Ubiquitin, and anti-β-actin (Sigma-Aldrich Corporation) antibodies.

### Immunhistochemistry

To detect cell proliferation in colon, paraffin-embedded colon and xenograft sections were deparaffinized. To unmask antigens, colon sections were boiled for 15 min in Antigen Unmasking Solution. Then colon sections were blocked using 5% goat serum in PBS and stained with a rabbit anti-ki67 monoclonal antibody (Biocare Medical, LLC) overnight at 4°C and followed by MACH 2 Universal HRP Polymer Detecion reagents (Biocare Medical, LLC) for 1 hour at room temperature. The sections were developed using the ImmPACT™ DAB substrate (Vector laboratories, Inc.), counterstained with hematoxylin, and observed using light microscopy. Ki67 positive cells in the colon tissues were determined by counting the absolute number of positive stained cells in at least 300 colonic crypts.

Immunostained xenograft slides were analyzed using the Ariol SL-50 automated slide scanner (Applied Imaging, San Jose, CA) to quantitate the number of ki67 positively stained cells. Cells were classified as positive or negative based on pre-determined thresholds that evaluate color and intensity of staining, as well as cell size, axis length, roundness, and compactness. Thus, blue hematoxylin staining of nuclei can be distinguished from brown DAB reaction product.

### Statistical analysis

Statistical significance for multiple comparisons in each study was determined by one-way ANOVA followed by Newman-Keuls analysis using Prism 5.0 (GraphPad Software, Inc.). A p value<0.05 was defined as statistically significant. All data presented are representative of at least three replicate experiments and are presented as mean ± S.E.M.

## Supporting Information

Figure S1 **Berberine regulates genes involved in proliferation and cell cycle arrest in IMCE.** Cells were treated with berberine at 50 µM for 24 hours and RNA was prepared for affymetrix expresion array. (A) Gene oncology classification of genes with the percentage is shown. (B) berberine-regulated expression of genes involved in cell proliferation and cell cycle arrest is shown. * *p*<0.01 compared to the control group. Data in this Figure are representative of two separate microarray assays.
(TIF)

Figure S2 **Berberine does not stimulates capase-dependent apoptosis in colon tumor cells.** Cells were cultured in serum-starved RPMI 1640 medium at 37°C for 24 hours with or without berberine at 50 µM treatment for indicated times or TNF (100 ng/ml) and cycloheximide (1 µg/ml) for 6 hours from the end of the experiment. Cellular lysates were collected for Western blot analysis to detect indicated signaling pathways. TNF and cycloheximide treatment was used as a positive control for induction of apoptosis.
(TIF)

Figure S3 **Inhibition of lysosome activity blocks berberine-induced EGFR down-regulation and inhibition of proliferation in colon tumor cells.** Cells were treated with berberine at 50 µM in serum-starved RPMI 1640 medium at 37°C for 18 hours with or without chloroquine at 30 µM. Cellular lysates were collected for Western blot analysis to detect indicated signaling pathways.
(TIF)

Material S1 (DOCX)
