# Supplementary figures and images for: Berberine Inhibits Proliferation and Down-Regulates Epidermal Growth Factor Receptor through Activation of Cbl in Colon Tumor Cells
Source: PLoS One. 2013 Feb 14;8(2):e56666. doi: 10.1371/journal.pone.0056666 (PMC3573001; doi:10.1371/journal.pone.0056666)

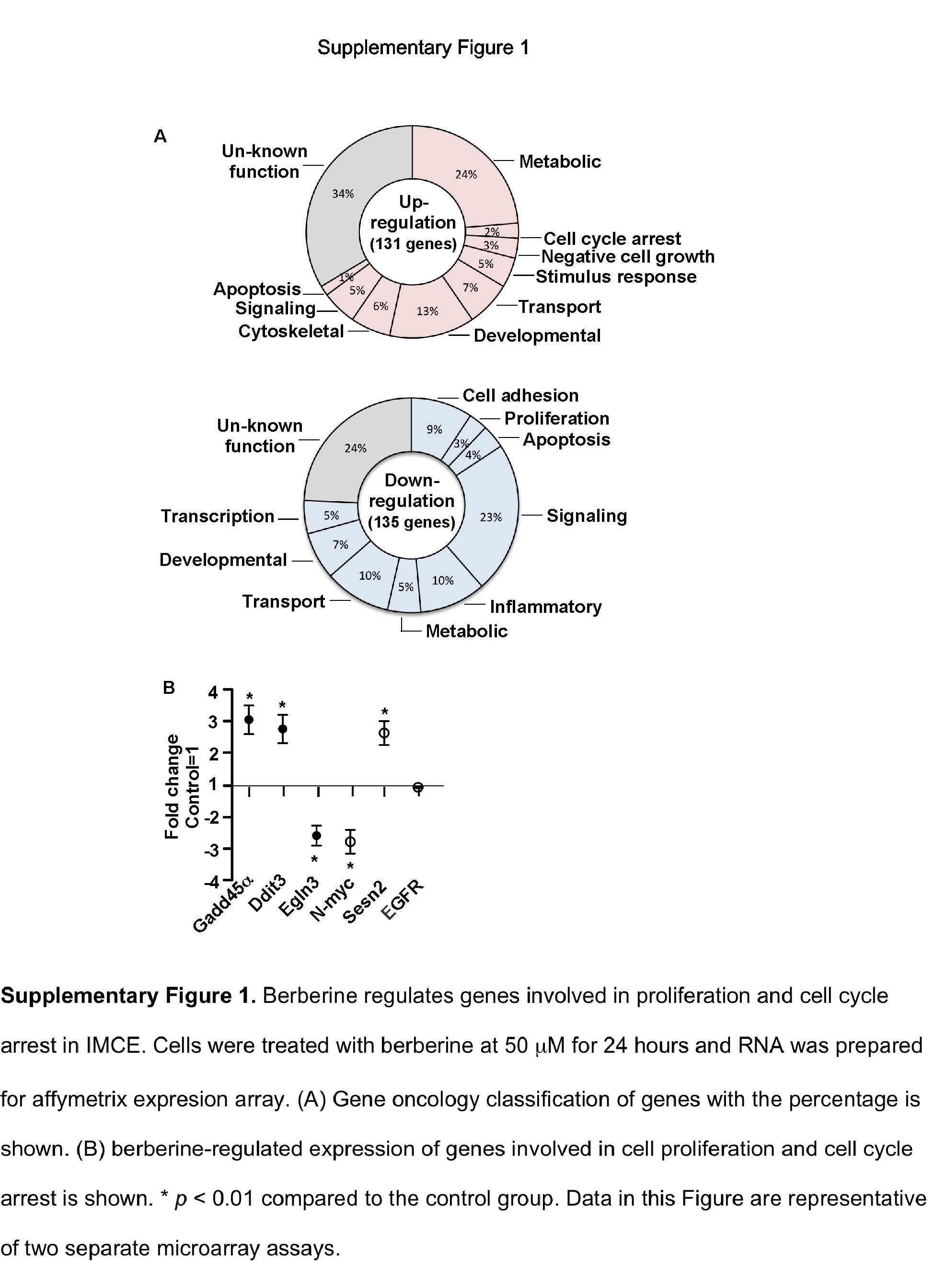

Supplement: Figure S1 — Berberine regulates genes involved in proliferation and cell cycle arrest in IMCE. Cells were treated with berberine at 50 µM for 24 hours and RNA was prepared for affymetrix expresion array. (A) Gene oncology classification of genes with the percentage is shown. (B) berberine-regulated expression of genes involved in cell proliferation and cell cycle arrest is shown. * p<0.01 compared to the control group. Data in this Figure are representative of two separate microarray assays. (TIF) [file pone.0056666.s001.tif]

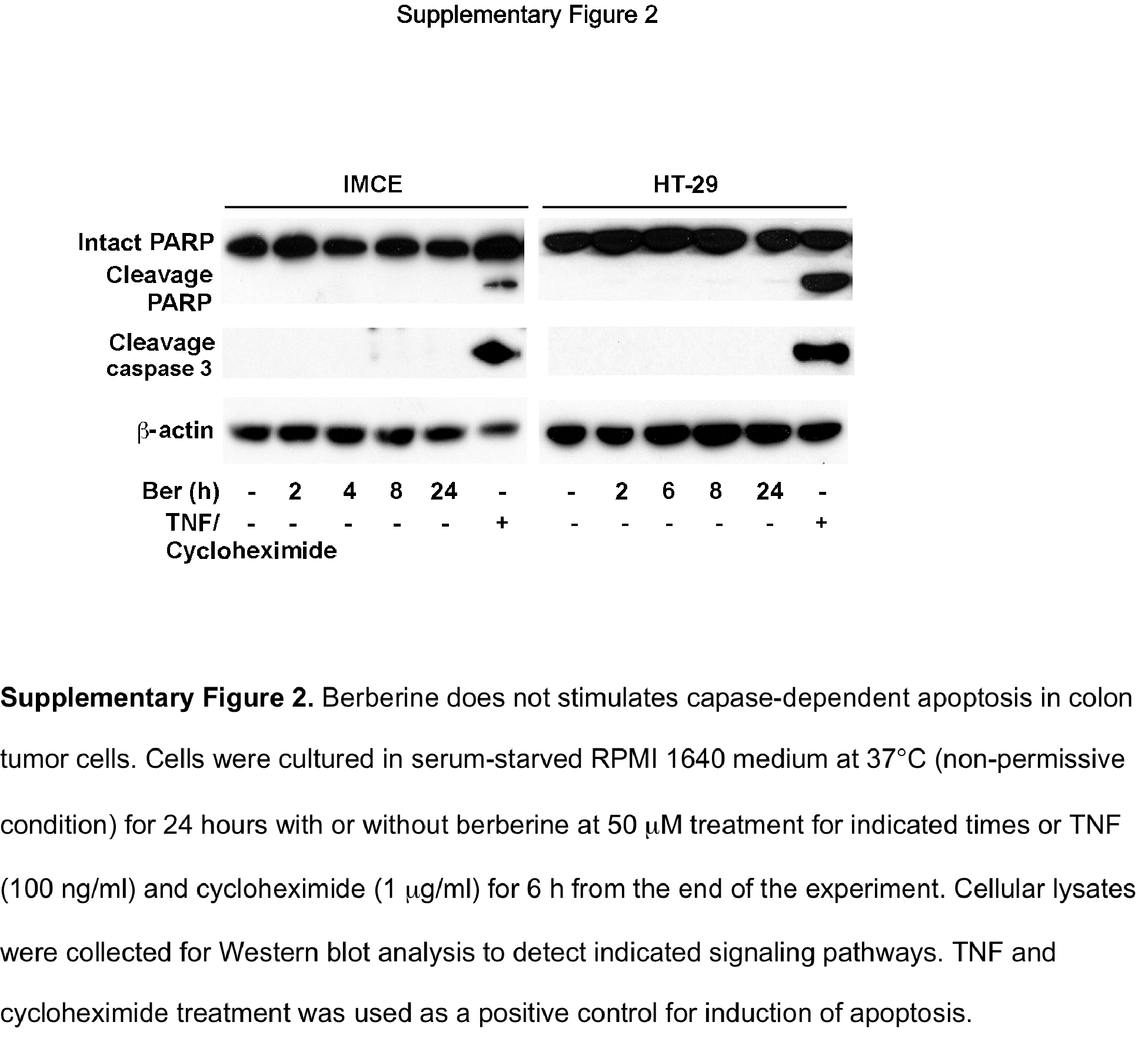

Supplement: Figure S2 — Berberine does not stimulates capase-dependent apoptosis in colon tumor cells. Cells were cultured in serum-starved RPMI 1640 medium at 37°C for 24 hours with or without berberine at 50 µM treatment for indicated times or TNF (100 ng/ml) and cycloheximide (1 µg/ml) for 6 hours from the end of the experiment. Cellular lysates were collected for Western blot analysis to detect indicated signaling pathways. TNF and cycloheximide treatment was used as a positive control for induction of apoptosis. (TIF) [file pone.0056666.s002.tif]

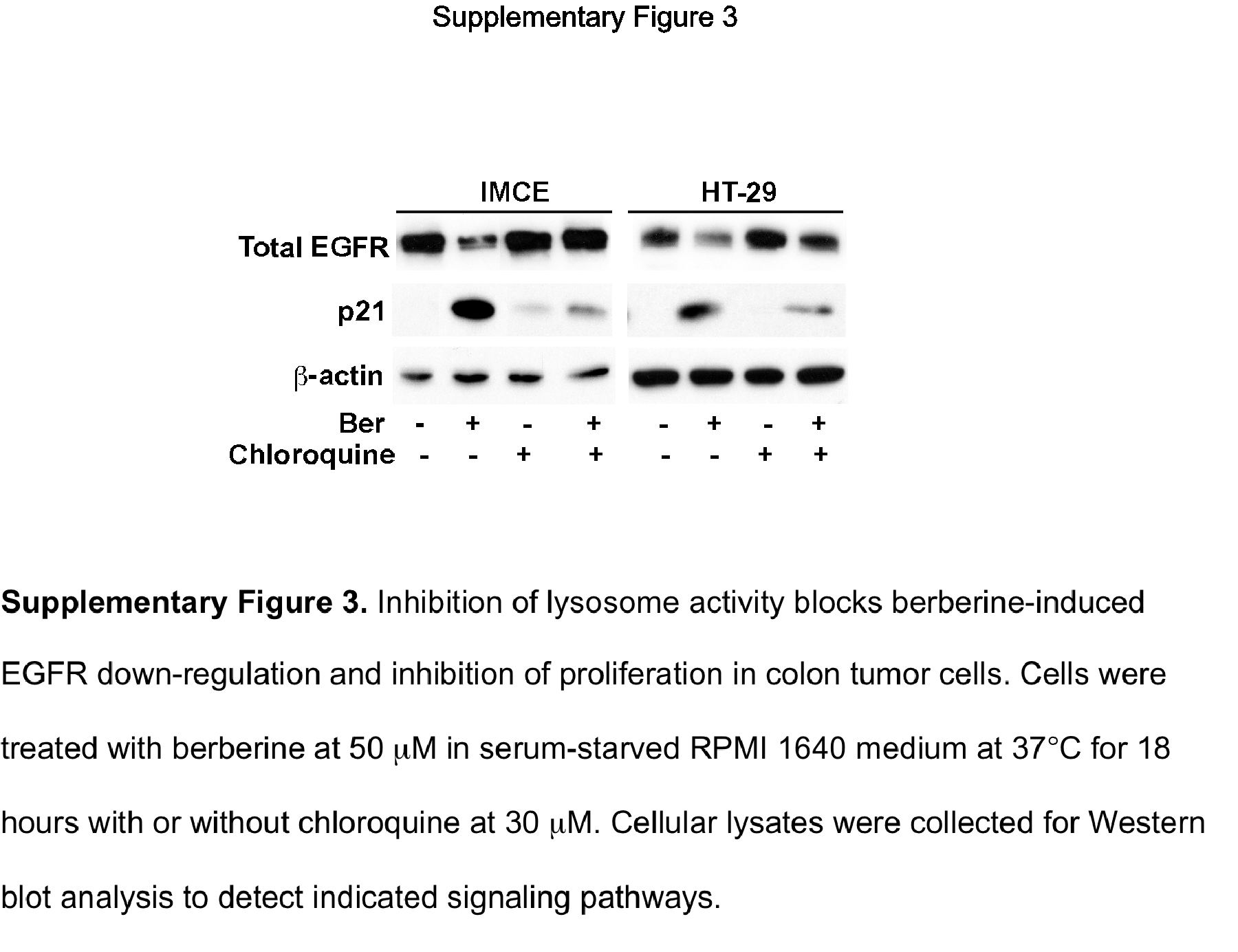

Supplement: Figure S3 — Inhibition of lysosome activity blocks berberine-induced EGFR down-regulation and inhibition of proliferation in colon tumor cells. Cells were treated with berberine at 50 µM in serum-starved RPMI 1640 medium at 37°C for 18 hours with or without chloroquine at 30 µM. Cellular lysates were collected for Western blot analysis to detect indicated signaling pathways. (TIF) [file pone.0056666.s003.tif]
