# Supplementary material for: Berberine Inhibits Proliferation and Down-Regulates Epidermal Growth Factor Receptor through Activation of Cbl in Colon Tumor Cells
Source: PLoS One. 2013 Feb 14;8(2):e56666. doi: 10.1371/journal.pone.0056666 (PMC3573001; doi:10.1371/journal.pone.0056666)
Supplement: Material S1 — (DOCX) [file pone.0056666.s004.docx]

**Methods**

Affymetrix microarray

RNA was isolated from IMCE cells in the presence or absence of berberine treatment. Mouse gene 1.1 ST Affymetrix GeneChip microarrays (Affymetrix, Foster City, CA) were performed using GeneTitan Instrument. Primary analysis of all expression data was performed using a combination of Affymetrix Expresion Console, comparisons using Biotique XRay, a Microsoft Excel plug-in, and NetAffx for gene ontology groupings.

**Results**

Berberine-regulated molecular consequences involved in proliferation in colon tumor cells.

To define the transcriptional regulation by berberine in the colon tumor cells that may be involved in cell growth, we analyzed RNA from IMCE cells with or without 24-hour berberine treatment by microarray. Using a requirement of minimum of a 2x change, a minimum expression intensity of higher of the pair of at least 128 (arbitrary affimatrix unites) and average expression values separated by more than the sum of their standard deviations (p<0.05 for difference), we found 131 (up-regulated) and 135 (down-regulated) unique genes with gene ontology information differentially changed by berberine treatment. Representative significant changes in gene expression regulated by berberine are shown (Supplementary Figure 1). Distribution of gene ontology groups up-regulated by berberine consist of metabolic (24%), developmental (13%), transport (7%), cytoskeletal (6%), signaling (5%), stimulus response (5%), negative regulation of proliferation (3%), cell cycle arrest (2%) and apoptosis (1%). Genes down-regulated by berberine include G-protein coupled receptor and cell surface receptor signaling (23%), inflammation (10%), transport (10%), cell adhesion (9%), developmental (7%), metabolic (5%), transcription (5%), apoptosis (4), proliferation (3%). We found congruent alterations in multiple cell cycle arrest and proliferation-related genes to the berberin’s inhibitory effects on cell proliferation and EGFR. Examples include up-regulation of two genes involved in cell cycle arrest, growth arrest and DNA damage-inducible 45 (Gadd45)α and DNA-damage-inducible transcript DDIT3, and a gene involved in inhibition of proliferation, sestrin (Sesn)2. Berberine also down-regulated N-myc, a protooncogene enhancing cell growth and proliferation (Figure S1).
